# Supplementary material for: Are carbon emissions trading and green financial instruments synergistic? -Comprehensive quantitative research based on content analysis
Source: PLoS One. 2024 Mar 7;19(3):e0298601. doi: 10.1371/journal.pone.0298601 (PMC10919601; doi:10.1371/journal.pone.0298601)
Supplement: S1 Data — (ZIP) [file pone.0298601.s001.zip › Data/地级市Z_F工作报告.zip!/╔╜╢1⁄2/║╩╘≤╩╨/└┤╫╘╣1⁄2╓┌║┼í╛┬φ┐╦╩2╛▌═°í┐_2005.docx]

各位代表:

　　现在,我代表市人民政府向大会作政府工作报告,请予审议,并请市政协委员和其他列席人员提出意见。

　　一、2004年工作回顾

　　过去的一年,在省委、省政府和市委的正确领导下,在“突破菏泽”战略的强力推动下,各级政府和全市人民高举邓小平理论和“三个代表”重要思想伟大旗帜,认真贯彻落实党的十六大和十六届三中、四中全会精神,以科学发展观为指导,正确把握国家宏观调控政策,从菏泽实际出发,创造性地开展工作,顺利完成了市十六届人大二次会议确定的各项任务,全面实现了“三个高于”的目标。

　　经济发展速度明显加快。牢牢抓住发展这个第一要务,全力以赴推动经济快速发展。全市实现生产总值365亿元,比上年增长17.1%,增幅比上年加快4.8个百分点,成为近二十年来经济发展最快的一年。地方财政收入17.2亿元,按可比口径增长31.4%。城镇居民人均可支配收入6734元,增长14.1%。社会消费品零售总额达到177.4亿元,增长15.1%。

　　经济运行质量显著提高。围绕建设“三大基地”,积极培植优势产业和骨干企业,加快发展民营经济,大力推进结构调整和经济增长方式转变,促进了经济运行质量效益的提高和经济结构优化。全市规模以上工业企业571家,新增213家,完成增加值84.6亿元,增长39%;实现利税17.5亿元,增长49.8%;工业经济效益综合指数145.1%,提高20.9个百分点;高新技术产值占规模以上工业产值比重提高到12%。全市注册个体私营企业11.1万户,注册资金87.9亿元,增长61.7%,上缴税金7.75亿元,增长47.5%。第二产业增加值占GDP的比重首次超过第一产业,三次产业比由上年的40:35.7:24.3调整到37.3:40.6:22.1。

　　农村经济发展呈现良好局面。认真落实降低农业税税率和粮食直补等一系列支农政策,积极推进农业结构调整,加大对农村基础设施和黄河滩区安全建设投入,组织开展大规模劳务输出,农业和农村经济出现重大转机,农民收入大幅度增长。粮食总产317.2万吨,增产17.6%;棉花总产27.4万吨,增产20.4%;新增造林120万亩;肉类总产45.6万吨,增长16.1%。新增有组织劳务输出29.3万人。农民人均纯收入达到2653元,增加441元,增长19.9%,是1997年以来增长最快的一年。

　　固定资产投资快速增长。发挥我市区位、交通、资源综合优势,以招商引资为重点,进一步加大了投资力度。全社会固定资产投资283.6亿元,增长75.1%;投资结构有了明显改善,工业投资161.3亿元,占总投资的比重达到57%;投资过千万元的项目达到678个,完成投资占总投资的45.8%。菏关高速、济菏高速先后开工建设,改建国省道公路145公里,新建乡村公路4544公里。巨野煤田龙固矿井、郭屯矿井等一批大型项目建设进展顺利。德商高速、东新高速和电源项目的前期工作取得重大进展。经济园区建设步伐加快,完成投资65亿元。

　　城市建设实现重大突破。新一轮菏泽城市总体规划顺利通过省政府组织的专家评审,编制完成了赵王河公园、环城公园等一批专业规划和详细规划。以城市土地经营为突破口,进一步加大了各类城市资产的经营力度。对经营性土地全部实行了招标、拍卖、挂牌出让,实现政府经营土地纯收益8.4亿元,其中市区收益5.2亿元。集中建设了一批重点项目,相继建成了污水处理厂和黄河净水厂,基本完成了环城公园、赵王河公园一期工程,对人民路和中华路等10多条道路进行了改造。广大城市建设者用自己的行动创造出了“知难而进,励志改革,艰苦创业,奋勇争先”的城建精神。启动了59个房地产综合开发项目和一批城市商业设施建设。同时,实施了3万棵大树进城、拆墙透绿等城市绿化亮化工程,建设街头绿地24处,新增绿地面积41万平方米,使城市功能明显增强,城市面貌明显改观。组建了城市综合执法队伍,完善了城市规划管理体制和环卫管理体制,城市管理水平明显提高。

　　对外经贸持续高速增长。适应国家出口退税机制改革,积极调整出口产品结构,改善外贸经营方式,努力扩大利用外资,开拓国外劳务市场,对外经贸实现重大突破。进出口总额8.3亿美元,增长166.2%,增幅居全省首位,绝对值上升到全省第12位,其中出口6.9亿美元,增长136.8%。出口过500万美元的企业达到35家。实际利用外资1.96亿美元,增长44%。国际劳务输出708人,增长109%。

　　各项社会事业全面发展。就业和社会保障工作进一步加强,城镇新增就业再就业8.8万人;加大社会保险金征缴力度,确保了养老金和失业保险金及时足额发放。社会救助体系日趋完善,灾区群众和特困群体的生产生活得到及时救助。黄河滩区移民迁建工作取得阶段性成果。疾病预防控制体系、传染病救治体系建设步伐加快,新型农村合作医疗试点顺利推进,市属公立医院产权制度改革实现了新突破。科技创新能力不断提高,取得市级以上重大科技成果86项,其中1项获国家科技进步二等奖。教育事业有了新发展,以县为主的农村义务教育管理体制进一步落实;高考本科录取人数首次突破万人大关;菏泽学院正式挂牌招生,结束了没有本科院校的历史;职业技术教育蓬勃发展。精神文明创建活动广泛开展,文化大市建设再创佳绩,《山东汉子》获得中国艺术节五项大奖。竞技体育实现新突破,1人荣获雅典残奥会冠军。计划生育、国土资源管理和环境保护工作明显加强。民族工作受到国家表彰。“平安菏泽”建设顺利推进。双拥工作深入开展。人防、气象、地震、广播电视、新闻出版、档案、史志、外事、侨务、老龄、妇女、儿童和残疾人等各项事业都取得新的成绩。

　　政府自身建设进一步加强。适应实施“突破菏泽”战略的需要,对干部作风进行了集中教育整顿。按照建立决策目标、执行责任、考核监督“三个体系”的要求,实行严格的目标责任制,并切实加强调度督查,推动了作风的转变,初步形成了一级抓一级、层层抓落实的工作机制。认真贯彻实施《行政许可法》和国务院《全面推进依法行政实施纲要》,深化行政审批制度改革,扩大政务公开,依法行政水平进一步提高。严格落实党风廉政建设责任制,对群众反映强烈的10个方面的问题进行纠风专项治理。市政府领导班子自觉遵守廉洁自律的各项规定,并向社会郑重做出廉政承诺,接受群众监督。

　　各位代表,过去的一年,是我市经济和社会事业发展取得重大成就的一年。这是省委、省政府“突破菏泽”战略强力推动的结果,是市委总揽全局、正确领导的结果,是人大、政协和社会各界有效监督、大力支持的结果,是全市人民团结拼搏、扎实苦干的结果。在此,我代表市人民政府,向全市工人、农民、知识分子和各级干部,向各民主党派、各人民团体和无党派人士,向驻菏部队、武警官兵和中央、省驻菏单位,向所有关心支持菏泽发展的国内外朋友,表示衷心的感谢!

　　回顾过去一年的工作,也使我们清醒地认识到,在经济社会发展中还有许多困难和问题,主要是工业化水平低,优势产业不明显,大型“龙头”项目少,园区发展滞后,企业融资渠道不畅;农村基础设施落后,农业产业化水平低,农民收入持续高速增长的基础还不稳固;社会保障体系不完善,救助困难群体的机制不健全,就业再就业的压力大;财源建设缓慢,财政收支矛盾突出,政府调控能力差,支持社会事业发展的财力匮乏。政府工作中还存在一些缺点和不足,有些政府工作人员依法行政意识不强,服务效率不高,工作作风不实。不作为、乱作为、失职渎职、贪污腐败的现象仍然存在。发展环境有待于进一步优化。

　　二、2005年目标任务

　　根据省委、省政府的要求和市委的部署,今年政府工作的总体要求是:以邓小平理论和“三个代表”重要思想为指导,认真贯彻党的十六大和十六届三中、四中全会精神,全面落实科学发展观,正确把握国家宏观调控政策,紧紧抓住“突破菏泽”的战略机遇,围绕实现“三三一一”目标,全力加快工业化和城市化进程,着力在招商引资和大项目建设、园区建设、城市建设上求突破,促进全市经济社会全面协调可持续发展。全市经济社会发展的主要预期目标是,生产总值增长15%,二、三产业比重提高3个百分点;全社会固定资产投资增长30%;地方财政收入增长15%;社会消费品零售总额增长15%;进出口增长25%,实际利用外资增长30%;农民人均纯收入和城镇居民人均可支配收入分别增长8%和10%;新增城镇就业8万人以上,城镇登记失业率控制在3.8%以内;人口自然增长率控制在6.9‰以内。实际工作中要加倍努力,力争发展得更快更好。

　　为实现以上目标,政府工作中要牢牢把握以下五条原则:

　　一是坚持把发展作为第一要务,抓住机遇加快发展。加快发展是解决一切矛盾的前提,是压倒一切的紧迫任务,是广大人民群众的根本利益所在。要紧紧抓住当前世界范围内产业转移的机遇、国家重点支持基础产业发展的机遇、省委省政府实施“突破菏泽”战略的机遇,始终把加快发展放在各项工作的首位,保护好、引导好、发挥好各方面干事创业的积极性,全力以赴推动经济社会快速健康发展。

　　二是坚持以结构调整为主线,加快推进工业化进程。工业化是改变我市欠发达面貌的治本之策,是“突破菏泽”、加快发展的根本出路。要围绕建设“三大基地”和培植“六大产业”,狠抓招商引资,扩大投资规模,通过加快工业化和城市化进程,实现以工促农、以城带乡,经济社会协调发展。

　　三是坚持以提高质量效益为中心,切实转变经济增长方式。

　　我市作为经济欠发达地区,要加快发展,必须按照科学发展观的要求,充分发挥后发优势,围绕提高质量效益,更加注重节约资源、降低消耗和保护环境,更加注重依靠科技进步和提高劳动者素质,推进经济增长方式从粗放型向集约型转变,走出一条科技含量高、经济效益好、资源消耗低、环境污染少、人力资源优势得到充分发挥的新型工业化道路。

　　四是坚持改革开放,增强发展活力。在认真贯彻国家宏观调控政策的前提下,大力推进市场取向的改革,利用市场手段配置资源,推动经济发展,解决社会矛盾,努力营造体制机制优势。坚持以开放促改革、促发展,紧盯经济全球化和区域经济一体化发展趋势,发挥优势,积极应对,大力发展对外贸易和国际经济技术合作,努力提高利用两种资源、两个市场的能力和水平。

　　五是坚持以人为本,促进经济社会协调发展。把维护和实现好群众利益作为一切工作的出发点和落脚点,在加快经济发展的同时,进一步加大对科技、教育、文化、卫生等各项社会事业的投入,做好与群众利益密切相关的工作,着力改善人民群众的生产生活条件,丰富群众的精神文化生活,创造有利于创业发展和人民安居乐业的良好环境,努力构建社会主义和谐社会。2005年,市政府拟着重抓好以下八项工作:

　　(一)以招商引资为重点,千方百计扩大投资

　　我市正处在工业化的起步阶段,加快发展主要靠投资。必须继续把扩大投资作为经济工作的总抓手,发挥综合优势,拓宽融资渠道,优化投资结构,实现投资新突破。

　　精心搞好项目策划。立足我市产业基础和资源优势,围绕建设“三大基地”和培植“六大产业”,依据国家产业政策和产业配套的要求,搞好产业策划和项目策划。按照投产达产一批、立项开工一批、策划储备一批的要求,始终保持1000亿元的项目储备,为招商引资、扩大投资创造条件。正确引导资金投向,避免低水平重复建设,提高投资效益。全力抓好招商引资。广泛开展“招商引资年”活动,动员全社会的力量招商引资。瞄准长三角、珠三角等产业资本聚集地区,集中组织招商。充分利用省委、省政府组织对口帮扶的机遇,大力推动“东企西进”。准确把握日、韩、港、台等国家和地区产业转移的趋势,组织好产业和项目对接,努力扩大利用外资规模。进一步改善招商引资方式,有针对性地开展登门招商、坐地招商、以商招商、委托招商,在抓好全民招商的同时,充分发挥专业招商队伍的作用,提高招商引资成效。继续办好牡丹花会和林产品交易会,更好地发挥招商引资平台作用。

　　积极争取政策支持。充分利用编制“十一五”计划的时机,努力做好项目前期工作,力争更多的项目列入国家和省计划,最大限度地争取国家和省政策性投资。对已经上报国家和省里的能源、交通等重点项目,组织专门力量靠上去做好争取工作,确保项目尽早开工建设。

　　全面激活民间资本。坚持把发展民营经济放在更加重要的位置,进一步放开经营范围,放开投资领域,消除各种歧视性与不合理规定,落实好扶持政策,优化民营经济发展环境,激发民间投资活力。加快建立融资担保、人才培训、技术信息、法规咨询等服务体系,促进民营经济快速发展。

　　加快经济园区建设。抓住东部八市在对口帮扶各县建设工业园区的机遇,创新园区投入方式,高标准进行园区基础设施建设,为项目进入创造良好条件。积极引导项目向园区集中,对入园项目进行严格把关,合理布局。搞好园区管理体制、运行机制和服务体系创新,实行封闭式管理,为投资者创造一个优质、高效的服务环境。举全市之力,办好市经济开发区,鼓励各县招商引资项目到市开发区落户,实现政策资源共享。

　　进一步优化投资环境。坚持市县乡主要领导以主要精力抓招商,并严格落实招商引资责任制和激励机制。完善招商引资政策规定,避免恶性竞争。抓住破坏投资发展环境的突出问题,集中力量进行专项治理。切实加大对“三难五乱”的整治力度,对各种行政不作为、乱作为,不但要追究直接责任者,而且要追究单位主要负责人的责任,情节恶劣的要公开曝光。对那些划地为牢、强装强卸的“市霸”、“村霸”、地痞流氓和各类黑恶势力,坚决予以严厉打击。

　　(二)围绕增加农民收入,加大“三农”工作力度

　　调整农业结构,增加农业收入。按照“五不准”要求,搞好耕地保护,在确保基本农田不减少的前提下,组织实施好中低产田改造,确保粮食单产提高、品质改善,总产稳定在300万吨以上。根据市场需求和当地优势,调整农业结构,大力发展果蔬、花卉等优质高效农业。创办农业示范园区,培植特色产业,提高农业产出效益。加快推进农业标准化步伐,建立健全农产品质量安全检测和动植物疫病防治体系,大力发展无公害农产品、绿色食品和有机食品,全市无公害农产品基地发展到200万亩。搞好滩区、沿路、沿河植树造林,并利用林地空间,做好林草牧结合文章,积极发展畜牧规模饲养,把菏泽建成全国重要的林牧生产基地。

　　发展农村二、三产业,增加非农收入。落实扶持政策,培植农业龙头企业,完善农村服务体系,拉长产业链条。积极发展各类农村合作经济组织,完善龙头企业与合作组织、生产基地及农户的利益联结机制,提高农业产业化经营水平。年内增加省级龙头企业4家以上,增加规模以上农产品加工企业60家以上。

　　抓好劳动力转移,增加工资性收入。适应我市工业和城镇快速发展的需要,抓好农村劳动力就地转移;继续组织好“西输东接”活动,促进农村劳动力向市外转移。以现有职业技术学校为依托,加大对农村劳动力的培训力度,以培训促转移。充分发挥市县两级劳动力市场的主渠道作用,加强对劳务中介组织的监管。实现农村劳动力的有序流动,全年新转移农村劳动力29.4万人,其中向市外转移17.4万人。

　　落实农村政策,促进农民增收。稳定和完善农村土地承包,引导规范土地流转。全面落实农业税税率再降低2个百分点等支农政策,确保土地纯收益的20%用于农业土地开发和基本农田建设。调整财政支出结构,加大对农业和农村的投入。加强政策督导,切实减轻农民负担。大力推进村务公开、民主理财、民主监督,依法维护农民的合法权益。

　　(三)着力培植“三个一批”,加快推进工业发展

　　按照培植“一批优势产业、一批骨干企业、一批名牌产品”的要求,围绕“六大产业”发展,突出抓好骨干企业膨胀。选择一批规模大、发展后劲足、优势明显的企业,在贷款担保、设备进口等方面给予更多扶持。继续深化产权改革,转换企业经营机制,增强内部发展活力。积极组织我市企业与国内外大企业、大集团挂靠、联合,借助外力加快发展。完善国资管理体制,落实国有资产经营责任制。高度重视破产重组企业的职工安置,维护企业和社会稳定。

　　推进企业技术进步。加快用先进适用技术和信息手段改造传统产业步伐,支持重大技改项目,全面提高产业技术装备水平,推动产业结构升级。加强企业技术创新体系建设,进一步提高企业自主创新能力,市科技三项经费用于支持骨干企业的比例不低于35%。大力发展高新技术企业,全市高新技术产值占规模以上工业产值的比重提高到14.5%以上。

　　实施品牌带动战略。选择一批具备一定基础、有发展前景的品牌,进行重点支持。同时,积极引进国内外知名品牌落户菏泽,借助外来品牌优势,带动我市相关产业的快速发展。

　　切实加强运行调度。加大对现有企业的支持力度,为企业发展创造宽松环境。及时帮助企业解决资金、原材料供应、市场开拓等方面遇到的困难,为企业健康运行提供方便条件。加强企业信用建设,加快建立信用担保体系,积极发展多元投资的担保机构,继续推进银企合作,争取金融机构的大力支持。

　　(四)以商贸流通为重点,促进服务业快速发展

　　从拉动经济增长、扩大劳动就业、培植地方财源的高度,把发展服务业放在更加重要的位置。加快专业批发市场建设。按照建设区域性商贸和物流基地的要求,根据菏泽城市总体规划和专业规划,搞好专业批发市场建设。加快恒盛物流大市场、施乐超市、康庄乐园服装大市场、鲁西南建材市场、鄄城中药材市场等大型商业设施建设步伐;抓好曹县桐木制品、郓城杨木制品以及成武大蒜,定陶、单县蔬菜,东明西瓜等专业市场的培育,着力提高辐射带动能力。全市专业批发市场交易额达到235亿元。

　　提升改造传统服务业。抓住我国服务业对外开放步伐加快和国内大型商业集团扩张的有利时机,采取加盟、连锁、特许经营等形式,积极引进市外资金和先进的管理理念、运作方式,提升传统服务业的档次和水平。整合物流资源,对现有仓储、运输、货代、联运企业进行重组改造,加快新型物流业发展。积极鼓励南华、鲁能、三信、华瑞等超市、连锁店向县乡延伸,建立城乡一体的销售网络。

　　加快发展新兴服务业。规范发展房地产、物业管理、社区服务业,积极培育会计、审计、咨询、法律、评估、拍卖等中介服务业,大力发展信息、休闲、保健、电子商务、金融保险等现代服务业。高度重视旅游业发展,加大旅游产品推销力度,着力打造菏泽牡丹旅游、历史文化旅游等特色旅游品牌,使其成为新的经济增长点。提升牡丹花会和林交会的策划、组织水平,推动会展经济发展。

　　继续扩大对外贸易。积极调整出口产品结构,在扩大果蔬、食品、裘革制品、木制工艺品等传统产品出口的同时,大力发展医药、化工、机电、纺织服装等产品出口。引导鼓励出口企业延伸产业链条,提高加工深度,增强市场竞争能力。改善外贸经营方式,以纺织、化工、木制品、搪瓷、造纸等五大行业为重点,大力发展加工贸易。发挥口岸直通优势,完善服务功能,扩大进出口货源,使之逐步成为辐射周边地区的进出口集散地。

　　(五)加快城市建设,创造优美人居环境

　　完善城市规划。以建设鲁苏豫皖四省交界处区域中心城市为目标,以城市总体规划为指导,完成开发区、老城区和城市中心区控制性详细规划,城市绿地、水系、防汛、市场、道路交通和管线综合等专业规划。严格规划程序,完善公众参与、专家评审和政府决策三位一体的规划审批机制,提高规划设计水平。规范城市规划管理,集中开展违法建筑治理活动,严厉打击乱占乱建行为。高起点搞好县城、重点建制镇和村庄建设规划,形成与中心城市功能互补、规模适度的城镇体系。

　　加快基础设施建设。加快建设菏关、济菏高速,开工建设德商、东新、济(宁)商高速,做好菏徐高速的前期工作,加快国省干线公路升级改造。完善黄河以桥代渡工程。做好洙水河航道改建工程前期工作,力争下半年开工建设。全面完成环城公园、赵王河公园一期配套建设任务,发挥治污、绿化、休闲和防汛调蓄为一体的综合功能。打通牡丹北路、和平南路,高标准改造牡丹路、黄河西路,翻修改造高平路、双井街等老城区背街巷道路。搞好“城中村”改造试点。高标准搞好牡丹路、黄河路等7条道路绿化,新建街头绿地20块。建成东、西两大水库,使城区水域面积达到8000亩。认真落实项目法人责任制,使之成为精品工程、廉政工程和民心工程。

　　加大城市经营力度。政府垄断城市土地一级市场,放开搞活二级市场,清理整顿隐形市场,对所有经营性用地坚持依法实行有偿出让。按照土地规划、征用、整理、出让、管理“五统一”的要求,搞好所有改造和新建道路两侧可利用地块的整体策划和开发招商,确保全市实现政府土地纯收益8亿元、市区5.2亿元的目标。全面推进城市有形资产和无形资产经营,增加政府收益。同时,积极利用国家开发银行的长期低息贷款,为城市建设提供资金保障。

　　提高城市管理水平。进一步加强社区建设,完善社区服务体系。巩固市区环卫体制改革成果,健全城市环卫管理机制。以车站、广场、干道、社区、绿地和公园为重点,集中开展市容市貌专项整治活动,打好“治乱”攻坚战。以车站、宾馆、商场、出租车等窗口单位为重点,广泛开展“做文明市民、树行业新风”活动,增强群众的城市意识,提高市民文明素质。发挥城市管理综合执法机构的职能作用,加大综合执法力度,形成依法管理和群众参与相结合的城市长效管理机制。

　　(六)做好财税金融工作,为经济社会发展提供切实保障

　　大力培植财源。准确把握国家财税政策导向,在推进工业化的进程中,着力加强财源建设,把经济发展的成果充分反映到地方财政收入和政府可用财力的增长上来。充分发挥财税杠杆作用,进一步加大对基础设施、支柱产业、骨干企业和城市建设等重点领域的支持,放大财政对促进发展、结构调整的导向作用。

　　强化税收征管。坚持依法治税、依率计征,按照税收不漏、不过、不拖的要求,全面推行税源精细化管理,加快税源监控体系建设。加强对重点税源企业和行业的监控,积极落实发票“双奖”制度,加快税控机的普及使用。加强税务稽查和监督,严厉打击偷逃骗税等涉税违法行为,维护公平竞争环境。完善部门协税工作责任制,加强社会综合治税,全面提高治税水平。

　　搞活国有资产经营。把行政事业单位和国有企业的所有经营性资产全部纳入市场化经营轨道,并实行有偿使用、目标经营,建立严格的监管体系,确保所有收益全部上缴财政。对非经营性国有资产,也要改革使用管理方式,最大限度地降低行政成本,堵塞漏洞。

　　严格支出管理。继续深化部门预算综合改革,完善和创新支出预算编制办法,严格按预算安排支出。在财政集中收付的基础上,实现会计委派和国库集中收付制度的并轨。进一步扩大政府采购范围,规范政府采购行为。优化财政支出结构,大力压缩会议费、接待费等一般性开支,严格控制车辆开支,确保工资及时发放,重点支持社会保障和农业、教育、科技、卫生等社会事业发展。

　　拓宽融资渠道。积极争取金融机构的支持。鼓励金融机构拓展金融产品,改进金融服务,增加信贷投放。加强金融监管,防范金融风险,深化金融改革,提高金融业经营管理水平。切实加快农村信用社发展,完善法人治理结构,不断壮大资金实力,更好地发挥为地方经济服务的职能作用。支持优势企业上市直接融资。

　　(七)大力实施“科教兴菏”和人才强市战略,加快各项社会事业发展

　　加快发展教育事业。进一步完善以县为主的农村义务教育管理体制,加大投入,强化管理,改善中小学办学条件,巩固发展“普九”成果;大力发展民办教育、职业技术教育和继续教育,办好菏泽学院,支持菏泽医专加快发展。加强素质教育,提高教育质量。坚决治理教育乱收费,切实减轻学生家庭负担。

　　大力促进科技进步。强化“一把手抓第一生产力”的意识,加快建设以产学研联合为主要形式的企业技术创新体系和以科技成果转化为重点的科技推广服务体系。建立多元化的科技投融资机制,多渠道增加科技投入。集中搞好影响经济社会发展的重大关键技术的开发引进,提高科技创新能力,不断增强科技对经济社会发展的支撑带动作用。

　　加强人才队伍建设。充分利用省支持我市引进人才的优惠政策,加快引进经济发展急需的高级技术和管理人才。高度关心和使用好现有人才,深化人事制度改革,建立鼓励优秀人才脱颖而出、人尽其才的有效机制和良好环境。

　　推进文化大市建设。加强对未成年人的思想道德教育,深入开展群众性精神文明创建活动,大力弘扬“菏泽精神”,不断提高全民思想道德素质。加强农村、社区、企业等基层文化建设。积极发展文学艺术、新闻出版和广播电视业。推动文化体制改革和机制创新,促进文化事业和文化产业共同发展。

　　支持卫生事业发展。加大政府投入,加快公共卫生体系建设;加强农村医疗卫生基础设施和农村医疗队伍建设,改善农村医疗卫生条件;搞好农村新型合作医疗试点,推进城镇医疗卫生体制改革;广泛开展爱国卫生运动。

　　发展各项社会事业。积极应对第四次人口生育高峰,全面落实计划生育基本国策,完善齐抓共管的合力机制,稳定低生育水平,提高人口素质。广泛开展全民健身活动,提高全民健康水平和竞技体育水平。关心支持老龄、妇女、儿童和残疾人事业,继续做好民族、宗教、档案、史志、外事、侨务、气象、防震减灾等工作,进一步搞好国防教育、人民防空和民兵预备役建设,做好转业军人、退役士兵安置和双拥工作,增进军政军民团结。

　　大力推进生态市建设。加大环境污染治理力度,加强重点流域、重点污染源的综合治理。严格执行环境影响评价和“三同时”制度,全面实施排污许可证制度,从源头上控制新的环境污染和生态破坏。加快各县污水处理设施建设。积极推行清洁生产,注重发展循环经济,努力建设资源节约型社会。加强对资源开发的监督管理,防止和遏制资源开发对生态环境的破坏。对煤炭、石油、天然气等不可再生资源,进行综合开发,持续利用,实现效益的最大化,决不能以破坏环境、浪费资源为代价换取一时的发展。

　　(八)坚持执政为民,真心实意解决关系群众生产生活的实际问题

　　牢固树立执政为民的理念,从关系群众切身利益的事情入手,竭尽全力为群众办实事、办好事,切实维护好群众利益。

　　积极扩大就业。坚持就业优先的原则,积极开发就业岗位,落实扶持政策,建立促进就业再就业的长效机制。完善市县两级劳动力市场,加快社区服务平台建设。力争年内开发和储备社区就业岗位3万个。全市生产自救基地由目前的27处发展到50处。

　　完善社会保障体系。以民营企业和灵活就业人员为重点,进一步加大社会保险扩面力度,确保把符合条件的各类城镇就业人员全部纳入社会保障范围。建立社会医疗救助制度,完善市级养老保险、失业保险调剂金制度。做好城市低保,实现动态管理下的应保尽保,逐步提高保障水平。关心城市低收入者的住房困难问题,市区开发建设廉租房、周转房400套,经济适用住房1000套。健全以“两证一卡”为主要内容的新的农村社会救济制度,帮助困难群众解决基本生活问题。积极发展慈善事业,推进社会福利社会化。

　　继续搞好农村基础设施建设。投资6.7亿元,新建农村公路4100公里,新增通柏油路行政村1027个;投资2400万元,新辟农村客运线路90条,新增通客车的行政村1258个,基本实现全市所有行政村村村通柏油路、村村通客车。投资7200万元,改造危桥167座。在全面解决历史性缺水人口饮水困难的基础上,尽快启动村村通自来水工程。积极做好滩区移民搬迁的后续工作,加快黄河滩区安全建设步伐。投资2.7亿元,完成农村电网改造三期工程。

　　整顿规范市场秩序。集中开展药品、食品等专项治理活动,严厉打击假冒伪劣、哄抬物价、强制消费、侵犯知识产权等不法行为,创造安全可靠的消费环境。加强社会信用体系建设,营造公平竞争、守法经营的社会环境,塑造菏泽诚实守信的良好形象。

　　继续推进“平安菏泽”建设。按照构建社会主义和谐社会的要求,以农村社会治安和社区治安为重点,进一步强化社会治安综合治理。认真做好人民来信来访工作,及时排查调处社会矛盾纠纷。高度重视安全生产,坚决杜绝重大安全事故和恶性事件的发生,保障人民群众生命财产安全。

　　三、加强政府自身建设

　　为切实履行好政府职责,圆满完成各项工作任务,市政府将按照“为民、务实、清廉”的要求,进一步加强自身建设,努力建设党和人民放心满意的政府。

　　加快转变政府职能。牢固树立发展是第一要务和以人为本、执政为民的执政理念,积极推进政府管理创新。按照“政企分开、政资分开、政事分开”的要求,进一步深化行政体制改革,切实把政府职能转到经济调节、市场监管、社会管理和公共服务上来,把政府经济管理职能转到主要为市场主体服务和创造良好发展环境上来,更加注重履行社会管理和公共服务职能,真正做到不越位、不错位、不缺位。从保障社会公共安全出发,加快建立健全各种灾害和突发事件应急机制,提高应对公共危机的能力。积极推进事业单位机构改革。继续抓好“政府提速”,完善行政审批中心的服务机制,方便人民群众,提高服务效率。

　　大力推进依法行政。认真贯彻实施《行政许可法》和国务院《全面推进依法行政实施纲要》,切实增强依法行政意识,严格按照法定权限和程序行使权力、履行职责,依法管理经济社会事务。坚持依法决策、依法办事、依法规范政府和公务员行为。深化行政执法体制改革,规范城市综合执法,解决多头执法和乱罚款等问题。加强行政执法监督,促进严格执法、公正执法和文明执法。实行执法责任制和执法过错追究制,完善并严格执行行政赔偿制度,做到有权必有责、用权受监督、侵权要赔偿。进一步完善群众参与、专家咨询和政府决策相结合的决策机制,提高政府决策的科学化、民主化水平。

　　切实加强政风建设。牢记“两个务必”,树立科学的发展观和正确的政绩观,弘扬求真务实精神,大兴求真务实之风,力戒官僚主义、形式主义,严禁劳民伤财的“政绩工程”、“形象工程”,坚决反对欺上瞒下、弄虚作假、虚报浮夸行为。深入开展“工作落实年”活动,建立完善决策目标、执行责任、考核监督“三个体系”,实行严格的目标责任管理,切实把各项工作落到实处、抓出实效。建立健全教育、制度、监督并重的惩治和预防腐败体系,严格落实党风廉政建设责任制,加强领导干部廉洁自律。坚决查处违纪违法案件,坚决惩处腐败分子,坚决纠正损害人民利益的不正之风。坚持从严治政,切实抓好党员先进性教育,加强公务员队伍管理,着力提高行政能力和水平,努力建设一支政治坚定、业务精通、清正廉洁、作风优良的公务员队伍。

　　自觉接受人民监督。政府的权力是人民赋予的,必须对人民负责,为人民谋利,受人民监督。只有人民监督政府,政府才不会懈怠。要自觉接受人民代表大会及其常委会的依法监督和人民政协的民主监督,广泛听取民主党派、无党派人士和工、青、妇等各人民团体的意见,认真办理人大代表议案、建议和政协委员提案。高度重视新闻舆论监督、社会公众监督和人民群众通过行政复议、行政诉讼等法定渠道对政府机关及其工作人员的监督。切实加强政府系统内部监督,支持监察、审计部门依法履行职责。实行“阳光政务”,扩大和规范政务公开,健全完善市长公开电话、政府网站、政策法规咨询中心和行政效能投诉中心的运行机制,增强政府工作透明度和社会公众参与度。

　　各位代表!新的一年,政府工作任务相当艰巨繁重。我们一定要增强责任感、紧迫感和危机感,团结带领全市各级政府,在中共菏泽市委的坚强领导下,与全市人民一道,以只争朝夕的精神和坚韧不拔的毅力,知难而进,扎实苦干,全力促进经济和社会事业更快更好地发展,确保实现“三三一一”目标,向全市人民交上一份满意的答卷!
